# Citizen Environmental Behavior From the Perspective of Psychological Distance Based on a Visual Analysis of Bibliometrics and Scientific Knowledge Mapping

**DOI:** 10.3389/fpsyg.2021.766907

**Published:** 2022-02-04

**Authors:** Li Yang, Xin Fang, Junqi Zhu

**Affiliations:** School of Economics and Management, Anhui University of Science and Technology, Huainan, China

**Keywords:** psychological distance, construal level, bibliometrics, environmental behavior, CiteSpace, VOSviewer

## Abstract

Global warming and other climate issues seriously threaten global sustainable development. As citizen environmental behavior can have a positive impact on the environment, it is of great theoretical significance and practical reference value to study the impact of psychological distance theory on citizen environmental behavior. This study obtained 2,633 related studies from 1980 to 2020 from the Web of Science as research objects, and used CiteSpace, VOSviewer, Netdraw, and other software programs to perform a bibliometric analysis, which can show the complex relationship implied in the citation, intuitively grasp the development context and hot frontier in this field, and help scholars better study citizen environmental behavior. The results and conclusions are as follows. (1) The related research covered three periods: infancy, growth, and outbreak. (2) Chronologically, the relevant research evolved from building models to analyze the impact of environmental enrichment on human environmental behavior, to studying the motivation behind citizen environmental behavior, and finally to paying attention to sustainable development. (3) A cluster analysis of high-frequency keywords identified three research hotspots: “drivers of psychological distance of environmental change perception on citizen environmental behavior,” “impact of social distance on adolescents’ behavior,” and “construal level theory and citizen green behavior.” Based on these findings, possible future research directions were identified, including changing from a single theory to a combination of multiple theories to comprehensively study citizen environmental protection behavior; analyzing the motivation of citizen environmental behavior and summarizing the general motivation of environmental behavior according to its internal relationship; and determining how to narrow the global psychological distance, strengthen the awareness of the community of human destiny, and explore the establishment of an efficient global climate cooperation mechanism.

## Introduction

Since the two industrial revolutions, countries worldwide have unilaterally pursued high-speed economic growth, placing a significant burden on the environment. Environmental problems have become a major challenge for sustainable development (Liao and Yang, 2021), and coordinating the balance between economic development and environmental protection has gradually become a global goal. British research on climate change perception has revealed that the public has a high awareness of the issue (Pidgeon, 2012), and most people believe that the world climate is changing and action should be taken to address it (Pidgeon et al., 2008; Garcia-Vazquez and García-Ael, 2021). Citizen environmental behavior has an extremely important positive impact on promoting environmental protection (Wibeck, 2014). Citizens highly agree with the concept of ecological civilization such as “lucid waters and lush mountains are invaluable assets,” and practice the concept of green and low-carbon consumption. At the individual level, the public can consciously protect the ecological environment, choose low-carbon travel modes, adhere to energy and resources conservation, and truly achieve a high degree of unity between environmental awareness and environmental behavior. At the social level, the public’s social responsibility has been continuously improved, actively respond to the social call, proactively participate in environmental protection voluntary activities, consciously participate in supervision and reporting, resist the behavior of non-environmental protection, pollution and waste, and contribute to the construction of ecological civilization. Environmental behavior, also known as pro-environmental behavior, was formally proposed by western scholars in the late 20th century. There is still a lack of a unified and recognized definition of environmental behavior, and we try to define it as the behavior of individuals actively participating in environmental protection in their daily lives. Environmental behavior has diversity and multi-dimensional structure. Pro-environmental behavior can include personal buying behavior, travel behavior, recycling and use of resources, and active participation in a pro-environmental organization (Snelgar, 2006); Hunter et al. (2004) classified environmental behavior into private environmental behavior and public environmental behavior according to the impact degree of environmental behavior; Larson et al. (2015) divided environmental behavior into four dimensions, namely, environmental lifestyle behavior, social environmentalism, environmental citizenship and land management; Tsai et al. (2021) divided environmental behavior into high-cost environmental behavior and low-cost environmental behavior from the perspective of cost; Gifford et al. subdivided the influencing factors of environmental behavior into 18 categories, namely, childhood experience, knowledge and education, personality and self-construction, sense of self-control, values, political attitude and world outlook, goals, sense of responsibility, cognitive bias, regional attachment, age, gender and regional differences, religion, urban-rural differences, social norms, social class, surrounding environment, cultural and ethnic differences (Gifford and Nilsson, 2014). At present, research concerning the motivation for environmental behavior mainly focuses on environmental attitudes, values (Mi et al., 2020), social media, and other factors. Thus, it is of great theoretical significance and practical reference value to use psychological distance theory to study citizen environmental behavior. The concept of psychological distance originated from Liberman and Trope’s time construction theory (Liberman and Trope, 1998, 2003) and later developed into the construal level theory, at which point psychological distance extended from time distance to space distance, social distance, and hypothetical research categories. Psychological distance refers to an individual’s perception of a distance in time and space and the possibility of occurrence of a described event or behavior with the self as the reference point. The event or behavior is distanced from the reference point in different ways, forming four dimensions: time distance, space distance, social distance, and hypothesis. Among these, space distance refers to an individual’s perception of the distance of space; time distance refers to an individual’s perception of the distance of the time of an event; social distance refers to the perception of the difference between the social object and self, and hypotheticality refers to the perception of an event or an object’s likelihood of occurrence or its distance from reality. Construal level theory states that time distance affects the interpretation level, thus affecting individual choices and decisions. In simple terms, the construal level can be considered to be people’s cognition and understanding of the objective world, which constitutes the basis of the evaluation and behavior results of a series of psychological distance events. Numerous studies have confirmed that interpretations will become more abstract with an increase in psychological distance, and that with an increase in interpretation level, the perception of psychological distance will also increase (Suzuki, 2019). Studies have shown that people traditionally believe that climate change risks only affect other people or countries and those born in the distant future. Risk perception is a strong driver of actions implemented to mitigate climate change (Osberghaus and Demski, 2019; Sheng et al., 2020), and effective perception involves belief in our individual or collective behavior ability, which is closely related to the assessment of the feasibility of mitigation measures (Roser-Renouf et al., 2014). Most people respond to climate change through personal experience, knowledge, a balance of benefits and costs, and trust in other social factors (Lorenzoni and Pidgeon, 2006). From the perspective of climate change communication, emphasizing the risk of climate change or the effectiveness of climate change mitigation may affect people’s support for mitigation policies or engagement in mitigation behavior. Wang et al. (2018) found that climate change as a serious threat to public health may provoke strong emotional reactions and promote people to participate in climate change mitigation. Fletcher et al. (2021) found 74% of responding Americans were concerned about climate change, however, when asked to describe travel in the year 2050 only 29% of participants discussed lower carbon options, suggesting that actively envisioning a sustainable future was less prevalent than climate change concern (Fletcher et al., 2021). In addition, emphasizing the ability of individuals or groups to contribute to climate change mitigation may increase participation in climate change mitigation (Morton et al., 2011; Ahn et al., 2015). On this basis, it has been found that information that emphasizes risk and effectiveness is more likely to motivate mitigation behavior than information that only addresses climate change risks (Xue et al., 2016). Narrowing the perception distance of climate change will lead to greater attention to climate change and more climate participation (Scannell and Gifford, 2013; Jones C. et al., 2017), immersive media can effectively bridge psychological distance (Fox et al., 2020; Breves and Schramm, 2021). Studies have found that communicating environmental risks will cause changes in individual behavioral intentions and behaviors (Bamberg and Schmidt, 2003; Sarid and Goldman, 2021; Smith et al., 2021). By publicizing environmental risks, people will have an increased awareness of environmental protection and change their attitude toward green behavior (De Groot and Steg, 2007). Environmental management is the collective responsibility of governments, individuals, and communities (Chen and Li, 2018), and many governments have recognized the importance of individuals in environmental management and have gradually incorporated individuals into the process of influencing their decision-making (Sobel, 2021). To achieve a sustainable improvement in urban air quality, citizens’ personal behavior, including the choice of daily transportation modes, must be altered to select environmentally friendly options (Chen et al., 2013).

Therefore, from the perspective of psychological distance, this study analyzes the motivation affecting citizen environmental behavior and examines the influence mechanism behind citizen environmental behavior. In recent years, researches on psychological distance theory and citizen environmental behavior have developed rapidly. However, there is a lack of literature reviews in this field, both locally and worldwide. Therefore, a scientific and quantitative analysis of the relevant literature on the impact of psychological distance theory on citizen environmental behavior will provide a reference for scholars to systematically study related development context and cutting-edge hot spots. This study used CiteSpace, VOSviewer, Bibexcel (Cheng et al., 2019), Netdraw (Shi et al., 2017), and other software programs to visually explore the relevant literature obtained from the core database of the Web of Science (WOS), comprehensively and systematically reviewed the research status of the impact of psychological distance theory on citizen environmental behavior from 1980 to 2020, excavated the development context and rising topics of interest, and proposed possible future research directions.

## Data Sources and Research Methods

### Data Source

**FIGURE 1:**
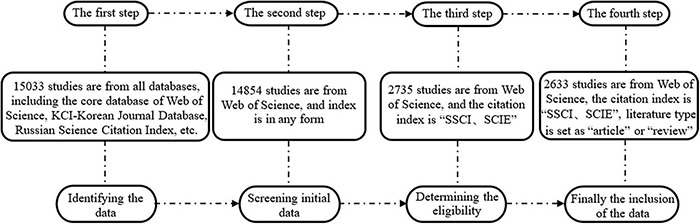
Identification process for relevant literature.

In this study, the core collection database of the WOS was selected as the literature source for data retrieval to ensure data reliability and authority. To further ensure the quality of the data, the citation index was set to “SSCI” or “SCIE,” and the literature type was set to “article” or “review.” The English search formula: TS = (“psychological distance” or “psychic distance” or “structural level” or “time distance” or “space distance” or “social distance” or “probability distance”) and (“environmental behavior” or “green behavior” or “pro-environmental behavior” or “environmental citizenship behavior” or “environmental organization citizenship behavior” or “low-carbon behavior” or “energy-saving behavior” or “employee green behavior” or “resilient behavior” or “sustainable behavior”) was employed for the period of 1980–2020. A total of 2,633 valid studies were obtained using the WOS literature output function and the CiteSpace deduplication function (see Figure 1 for specific steps).

### Research Methods

Bibliometrics is a discipline that studies the distribution structure, quantitative relationship, change law, and quantitative management of literature and information and then explores certain structures, characteristics, and laws of science and technology using a literature system and bibliometric characteristics as the research object, and employing the metrological research methods of mathematics and statistics (Guler et al., 2016; Bornmann and Marewski, 2019). A co-word analysis is a bibliometric analysis method derived from the concepts of citation coupling and co-citation in bibliometrics and has been widely used in metrological research, as it can describe and explain the knowledge organization of a discipline (Feng et al., 2017; Zhu and Zhang, 2020). CiteSpace is a visual network analysis software based on citation analysis theory and the Java environment. It has been gradually developed under the background of scientometrics, data, and information visualization (Chen, 2006; Liu et al., 2015), showing the complex relationship implied in the citation. VOSviewer is a bibliometric analysis software based on the principle of co-citation, and is used to draw a map of scientific knowledge in various fields of knowledge (Waltman et al., 2010). In this study, the data collected were analyzed visually using the WOS data analysis module in CiteSpace5.5.R2. The obtained relevant information, such as the number of articles published year by year, issuing institutions, and source countries, were objectively evaluated considering the research status of the impact of psychological distance theory on citizen environmental behavior. Then, VOSviewer was used to generate a “keyword co-occurrence network” and “keyword time zone map” to evaluate the research hotspots and evolution context in this field. Finally, the results of the bibliometric analysis were summarized, and future research directions were discussed.

## Visual Analysis of Knowledge Map

### Analysis of Time Characteristics of Posting Volume

**FIGURE 2:**
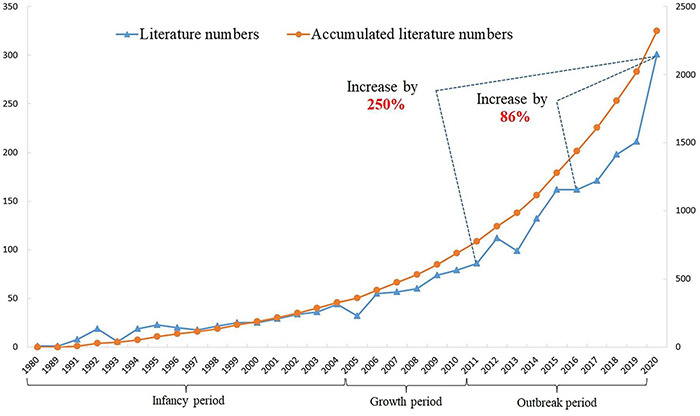
Annual numbers and growth rates of citizen’s environmental behavior related literature.

The number of articles published on a specific topic in international academic journals represents, to a certain extent, the degree of concern for the research topic. Meanwhile, the data of an annual publication volume and its growth rate can reflect the rise time of the research topic and the change in its degree of concern. Figure 2 shows that the earliest article of citizen environmental behavior from the perspective of psychological distance was published in 1980. During the 45 years from 1980 to 2005, the average annual number of articles published was less than nine, which is low. We defined this period as the infancy of the impact of psychological distance theory on citizen environmental behavior. From 2006 to 2011, the annual number of articles published began to exceed 50 and increased annually, and this time period was thus defined as the steady development period of the study. Since 2012, the number of research papers has exceeded 100 each year, showing a rapid growth trend. In 2020, the number of articles exceeded 300 for the first time, which was defined as the outbreak period. In the past decade (2011–2020), the number of articles published increased by 250%, showing an explosive growth trend, and in the past 5 years (2016–2020), the number of articles published increased by 86%, showing a rapid growth trend.

### Distribution of Issuing Countries and Journals

A further analysis of the distribution of countries that published articles showed that 116 countries have conducted research regarding the impact of psychological distance theory on citizen environmental behavior. The United States produced the most research in the field of psychological distance theory on citizen environmental behavior, with 839 papers published in this field, accounting for 31.86% of the total number of papers. Meanwhile, China and Great Britain ranked second and third, respectively, with 268 and 258 papers, accounting for 10.18 and 9.80% of the total, followed by Germany, France, and Canada. The top 20 publishing countries are listed in Table 1.

**TABLE 1:** Distribution by country of literature on citizen environmental behavior (top 20).

Country	*N*	*P*	Country	*N*	*P*	Country	*N*	*P*
United States	839	31.86%	Italy	136	5.17%	South Africa	56	2.13%
China	268	10.18%	Spain	132	5.01%	Norway	55	2.09%
Great Britain	258	9.80%	Netherlands	115	4.37%	Belgium	52	1.97%
Germany	213	8.09%	Japan	94	3.57%	New Zealand	51	1.94%
France	185	7.03%	Brazil	93	3.53%	Portugal	46	1.75%
Canada	184	6.99%	Switzerland	81	3.08%	India	45	1.71%
Australia	178	6.76%	Scotland	74	2.81%			

Understanding the journal distribution of citation literature can help researchers determine research hotspots and frontiers in this field *via* appropriate journals. The distribution of journals with 15 or more papers in the WOS core collection is listed in Table 2. Relevant research has been published in ecology, environmental science, zoology, behavioral science, environmental research, and other related journals. Specifically, PLOS ONE published the most studies (57 papers), followed by APPLIED ANIMAL BEHAVIOR SCIENCE (38 papers), SUSTAINABILITY (35 papers), and ANIMAL BEHAVIOR (33 papers). These findings show that research on the impact of psychological distance theory on citizen environmental behavior is rich.

**TABLE 2:** Journal distribution of literature on citizen environmental behavior (top 20).

Journal	*N*	Journal	*N*
PLOS ONE	57	Ecology and evolution	18
Applied animal behavior science	38	Journal of experimental biology	18
Sustainability	35	Frontiers in psychology	17
Animal behavior	33	International journal of behavioral nutrition and physical activity	17
Marine ecology progress series	30	Behavioral ecology	16
International journal of environmental research and public health	25	Journal of ornithology	16
Journal of animal ecology	24	Journal of transport health	15
Scientific reports	23	Marine biology	15
Science of the total environment	22	Transportation research part d transport and environment	15
Behavioral ecology and sociobiology	21	Urban forestry urban greening	15

### Literature Co-citation Analysis and Knowledge Base Identification

**FIGURE 3:**
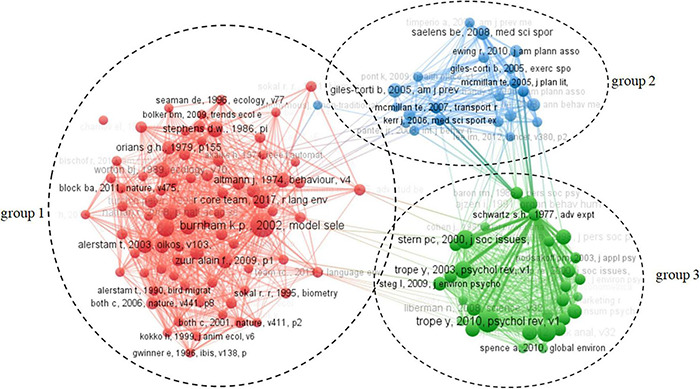
Visualization atlas of literature co-citation network.

Literature co-citation analysis is a research method to measure the degree of relationship between literature, exploring the development and evolution. There were 108,940 citations in 2,633 articles on citizen environmental behavior from the perspective of psychological distance collected in this study; the minimum number of citations was set as 13, and 110 citations were finally obtained. For the 110 selected studies, the modular layout and clustering method of VOSviewer were used to construct a visual map of the literature co-citation network, as shown in Figure 3.

In Figure 3, 110 nodes represent 110 studies, and the distance between nodes represents the similarity of the literature. The visualization results showed that 110 citations were automatically divided into three clusters according to the VOSviewer clustering method. The knowledge base of citizen environmental behavior was divided into three knowledge groups by merging and summarizing the topics of every cluster: influencing factors of citizen environmental behavior (knowledge group 1), conceptual framework and theoretical models (knowledge group 2), and citizen environmental behavior in response to climate change (knowledge group 3).

Knowledge base 1: influencing factors of citizen environmental behavior.

Knowledge group 1 focused on influencing factors of citizen environmental behavior. In order to effectively deal with climate change, many scholars have carried out researches on citizen environmental behavior. Neves and Brand (2019) collected travel data of some residents through observation and research, and found significant potential of active travel to substitute short car trips, with sizeable impacts on carbon emissions from personal travel, which benefits improving air quality, reducing noise and fossil fuel use. Car use in urban areas carries well-known risks for negative effects on urban quality of life, the environment and land use. Pro-environmental attitudes and personal norms affect the habit intensity of automobile use. In order to reduce the formation of strong automobile habit intensity, we should carefully deal with demographic, spatial and psychosocial factors (Şimşekoğlu et al., 2017). The energy consumed by the construction industry accounts for 40% of the global energy consumption, which is seriously affecting our living environment (Bang et al., 2021). Zhang et al. (2019) studied the motivation of buyers’ green purchase behavior and stimulated consumers’ market demand for green products. Mir et al. (2016) evaluated the impact of air pollution result framework and psychological distance on citizen willingness to environmentally friendly behavior, especially the choice of changing travel mode. The results show that the consequences of spreading air pollution will encourage individuals to take more environmentally friendly actions and change their intention to use more sustainable transportation modes. Travelers environmental awareness will affect their mode choice, Bai et al. (2020) found that original car users tend to choose medium or zero emission mode; original bus and subway users tend to choose zero emission mode or original mode; few initial cyclists and walkers prefer medium or high emission mode. The study results provide insights for the transport sector to establish sustainable urban traffic management policies and strategies to increase the share of zero emission and low emission modes of transport (Park et al., 2020).

(2) Knowledge base 2: conceptual framework and theoretical models.

Knowledge group 2 focused on conceptual framework and theoretical models. Li et al. (2021) were the first to study the framework effect, using perceived value and pro-environmental personal norms to explain citizen environmental behavior. Bamberg et al. (2007) put forward a comprehensive theoretical framework to discuss the role of personal norms in deciding to use public transport, and empirically explained how different social environments affect citizen choice of travel modes. Jones C. et al. (2017) used explanatory theory to study whether climate change communication intervention can increase public participation by shortening the psychological distance of climate change, the results showed that climate communication aimed at shortening the psychological distance is an effective strategy to increase public participation in climate change. Amatulli et al. (2019) found that negatively framed messages are more effective than positively framed ones in prompting consumers to engage in pro-environmental behaviors (Amatulli et al., 2019). Huang (Huang et al., 2021) introduced the theory of planned behavior, which provided a useful conceptual framework for solving the complex problems in human social behavior. Gifford (2011) found that most people believe that climate change and sustainability are very important, but their pro-environmental behavior is often limited due to seven types of psychological barriers. Dunlap et al. (2000) revised the new environmental paradigm scale, which can better explore the key aspects of ecosystem. Leiserowitz (2005) found that Americans believe that climate change is a medium risk, which will mainly affect people and places far away in geography and time. Public risk perception can fundamentally force or restrict political, economic and social actions to deal with specific risks.

(3) Knowledge base 3: citizen environmental behavior in response to climate change.

Knowledge group 3 mainly focused on citizen environmental behavior in response to climate change. Mcdonload (McDonald et al., 2015) found that personal experience related to climate change can often potentially affect people’s environmental behavior, but not reducing psychological distance will always lead to more attention or action on climate change, and even face potential risks of fear and avoidance. Wu et al. (2014) believed that the construction industry can effectively respond to global climate change by formulating carbon labeling schemes, and change consumers’ purchase behavior by labeling low-carbon products, so as to help the construction industry develop into a green industry. Ye et al. (2021) examined the regulatory role of visual cues in promoting energy conservation from a new perspective, which show that the persuasiveness of descriptive normative appeal tends to be more robust than injunctive appeal, descriptive and prohibitive normative appeal can positively affect the actual energy-saving behavior. Ho et al. (2020) believed that the low level of public participation in climate change may be due to the neglect of the future threat of climate change and the distant benefits of mitigation behavior, the research found that raising awareness of climate change can promote pro-environmental behavior. Social norms arised from the expectation of others’ behavior and the consequences of abiding by or deviating from these norms, meta-analysis showed that social norms can promote behavior conducive to the environment (Perry et al., 2021). Gao et al. (2021) analyzed the relationship between public awareness of green products and price sensitivity, and results showed that public recognition, self-explanation and time distance played an important role in predicting the price sensitivity of green products. Yan et al. (2021a) discussed how the psychological feeling of power affects consumers’ preference for green products, and found that the influence of power on green consumption is more prominent among the people with high green consumption value.

### High Frequency Keyword Feature Analysis

Keywords can reflect the main content of an article well and are highly generalized and concise regarding the research topic. High-frequency keywords usually reflect current issues and frontier trends in a specific research field. Therefore, a statistical analysis of literature keywords can quickly and effectively identify research hotspots. In this study, Bibexcel was used to analyze keywords in the published literature using keyword frequency analysis technology. However, this software cannot accurately identify word abbreviations or singular and plural words and has other issues. Therefore, we invited five graduate students and two professors from relevant research directions to integrate the keywords. For example, “LOCOMOTION,” “BIRD MIGRATION,” “PARTIAL MIGRATION” and “ANIMAL MIGRATION” were merged into “MIGRATION,” and “TELEMETRY” was merged into “SATELLITE TELEMETRY.” After this integration process, keywords with frequencies of ≥ 16 were selected as high-frequency keywords for further analysis. Table 3 lists the top 20 high-frequency keywords.

**TABLE 3:** High-frequency keywords of literature on citizen environmental behavior (top 20).

Rank	Keywords	Frequency	Rank	Keywords	Frequency
1	Behavior	623	11	Population	82
2	Model	143	12	Response	79
3	Climate change	139	13	Built environment	74
4	Pattern	130	14	Perception	66
5	Physical activity	125	15	Performance	66
6	Movement	101	16	Conservation	54
7	Impact	99	17	Risk	49
8	Environment	85	18	Psychological distance	47
9	Ecology	84	19	Attitude	41
10	Health	84	20	Green	31

The 60 × 60 co-occurrence matrix generated by Bibexcel was imported into Ucinet (Xue et al., 2020) to generate a file, which was read by Netdraw to draw a social network map of highly cited keywords in the study of citizen environmental behavior based on psychological distance theory (Figure 3). In the map, each high-frequency keyword represents a node, and the connecting line between two nodes represents the relationship between them. The size of the node in the figure represents the degree of centrality, wherein the larger the size, the larger the connection range, and the greater amount of collinear relationships with other keywords. Through an additional analysis of the high-frequency keywords, we summarized the literature topics of each cluster and obtained three research hotspots in the research field of citizen environmental behavior: drivers of psychological distance of environmental change perception on citizen environmental behavior (hotspot 1), including behavior, cognition, environmental change, environmental enrichment, habitat fragmentation, and other keywords; the impact of social distance on adolescents’ behavior (hotspot 2), including adolescents, built environment, social environment, and physical activity; and the construal level theory and citizen green behavior (hotspot 3), which include attitudes, climate change, construal level theory, environmental behavior, and sustainability.

(1) Research focus 1: drivers of psychological distance of environmental change perception on citizen environmental behavior.

Hotspot 1 focused on the drivers of psychological distance of environmental change perception on citizen environmental behavior. Western society has become increasingly concerned about the harm caused by some products to the environment, and many consumers have begun to change their purchasing behavior and support producers selling organic products or participating in green policies (De Maya et al., 2011; Tong et al., 2020). De-Magistris and Gracia (2016) used a non-hypothetical evaluation method to simulate the real market with real products and money and found that consumers were more willing to accept sustainable consumption in order to reduce greenhouse gas emissions. Climate change is one of the most serious health threats in the 21st century and will affect population groups differently depending on their geographical location, age, ethnicity, health status, and socioeconomic circumstances. New Zealand is a typical climate country, wherein more than half of the public is very concerned about climate change and hopes to reduce emissions by implementing individual scale actions (Hopkins et al., 2015). Plastics and other man-made wastes pose a highly pervasive and persistent threat to the global marine ecosystem. Nelms et al. (2017) conducted several citizen science projects, which made positive changes in citizen behaviors and attitudes and raised their awareness of environmental issues. Moreover, policies to phase out fossil fuel vehicles are key to avoiding dangerous and irreversible global climate changes, but a survey found that perceived psychological distance between climate change and political party identity influences policy preferences (Rinscheid et al., 2020). Feng et al. (2021) found psychological distance plays a greater role than price in the long-term purchase of green housing. To sum up, we found that citizen psychological perception of environmental change can directly affect citizen environmental behavior. Researches show that most citizens actively or passively participate in environmental behavior, including buying green products and using green travel modes, in order to reduce damage to the environment and slow down the rate of climate deterioration. At the same time, there is also a minority of people, Although they perceive the psychological distance of climate change, they are still unwilling to engage in environmental behavior, which needs to continue to explore the in-depth motivation behind citizen environmental behavior in the next research.

(2) Research focus 2: impact of social distance on adolescents’ behavior.

Hotspot 2 focused on the impact of social distance on adolescents’ behavior. Verhoeven et al. (2016) investigated the factors that affect adolescents’ choice of active or passive transport and found social norms and social support contribute largely to active transport selection. In the micro sense, there are objectively different social distances among people with different origins, experiences, cultures, and professional backgrounds. Havdal et al. (2021) found that adolescents from families with lower socioeconomic status tend to have more unhealthy eating behaviors than those from families with higher socioeconomic status, which provides a solution to the social inequality in adolescents’ dietary behavior. The impact of cultural adaptation on social relations spans all levels of the social ecological framework, and adolescents in multicultural families face a variety of stressors, of which social support and community awareness significantly and directly affect college students’ well-being, and economic status and school type have an indirect impact on well-being (Shin et al., 2021). Corner et al. (2015) found that values and message framing can significantly affect young people’s participation in climate change action (Corner et al., 2015). De Rosis et al. (2020) believed that environmental factors affect adolescents’ behavioral preferences. For example, adolescents living near green areas tend to select more active and healthier green travel modes. Conversely, those living in industrial or heavily trafficked areas prefer to use motor vehicles. This finding is conducive to promoting healthy environmental behavior choices among adolescents. In conclusion, we must further explore how social distance affects adolescents’ behavior. Macro social distance includes historical tradition and cultural background, and adolescents’ behavior varies among different nationalities and countries; In the micro sense, adolescents from different backgrounds, experiences and majors behave differently, but they are a little similar. Social distance is often caused by objective environment and human factors. For human social distance, we can narrow psychological distance through future work, such as publicizing the severe harm of climate change and improving adolescents’ professional quality, these measures can effectively have a positive impact on adolescents’ environmental behavior.

(3) Research focus 3: construal level theory and citizen green behavior.

Hotspot 3 mainly focused on construal level theory and citizen green behavior. Mir et al. (2016) evaluated the impact of the interaction between the result framework and psychological distance of air pollution on citizen willingness to be environmentally friendly and found that communicating the consequences of air pollution can encourage individuals to adopt more environmentally friendly behavior, while reducing the psychological distance of air pollution to make the manipulation framework more personally relevant had no significant impact on the respondents. Advocating low-carbon travel and building sustainable low-carbon cities are the main trends of urban development. The urban space environment can lead to changes in residents’ travel mode, and resident travel will also have an impact on urban space layout. Zhou et al. (2019) studied the social attributes of passengers and the psychological threshold of different travel purposes to provide a reference for exploring the coupling degree between land utilization and resident demand. Yan et al. (2021b) studied the impact of social class on green consumption based on the optimal distinctiveness theory, and found the middle class has a greater tendency of green consumption compared with the lower and upper classes. Yu et al. (2017) used structural equation modeling to reveal that individuals must have environmental ethics, clear social and individual responsibilities, and consider time and space distance simultaneously to reduce the psychological distance of climate change and exhibit green environmental behavior. Hwang and Lee (2018) found that self-construction has a significant impact on ecological beliefs, which are significantly related to the satisfaction of ecotourism services that affect ecotourism behaviors. To lessen the environmental consequences of private car ownership, the government can introduce a feebate system, a CO2 emission VAT system and a distance based user charging scheme, which can incentivize the purchase of low emission vehicles (Carreno et al., 2014). Construal level theory originated from the time explanation theory and was first used to explain how people evaluate and plan the future. Later, it gradually developed into the impact of construal level on cognition and behavior. For example, when people realized the serious consequences of climate change, they will evaluate their personal behavior and decide whether to make behavior conducive to the environment, or influence others to make similar actions through their own personal efforts. The basis that citizens explained and evaluated the future often explains citizens green behavior.

### Analysis of Evolution Trend

**FIGURE 4:**
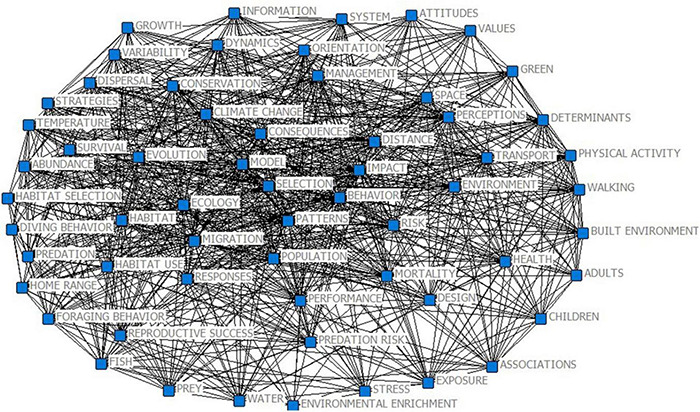
Social network diagram of high-frequency keywords.

CiteSpace can draw a time zone map of keywords according to a time series, which can intuitively present the hotspot distribution in every time period. Emerging words refer to keywords whose frequency increases rapidly within a certain period and can be used to predict emerging trends in this field. The combination of a CiteSpace time zone map and detecting emerging words can clearly show the evolution of a research topic. In this study, the top 50 most frequently used keywords (slice length = 2) from 1992 to 2020 were selected to build the keyword co-occurrence network, and a path-finding network was used to cut the merged network. The results are shown in Figure 4. The size of the node circle in the figure represents the frequency, and nodes with a purple outer circle exhibit high intermediary centrality, meaning they are hub nodes in the network, also known as turning points.

**FIGURE 5:**
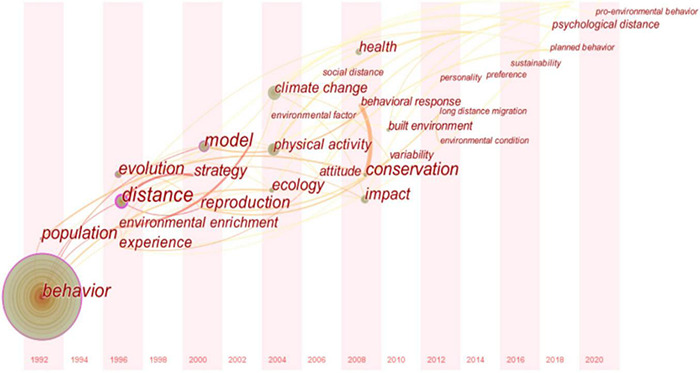
Keyword time zone evolution map.

(1) Before 2004, the research focused on building models to analyze the impact of environmental reproduction on human environmental behavior in order to achieve strategic objectives, such as improving the environment. This research divided environmental behavior into three categories. The first category is environmental behavior on an individual basis, that is, green consumption and lifestyle based on individuals or families, including the use of clean energy, purchasing of green food, and waste classification. The second category is the public environmental behavior of active participation, which refers to citizens consciously and actively supporting the government’s environmental policies and promoting environmental governance, including undertaking higher environmental taxes, supporting and implementing relevant environmental laws and regulations, and supervising enterprises to exhibit environmentally friendly production behavior. Finally, the third category is radical public environmental behavior, which refers to participating in environmental protection in a collective and confrontational way, including petitions, marches, demonstrations, and other activities for environmental protection (Pahl et al., 2014).

(2) From 2004 to 2015, with the deterioration of the environment and climate, an increasing number of scholars began to pay attention to psychological distance theory to study the motivation behind citizen environmental behavior and what behavioral measures citizens will employ to protect the ecological environment in the process of urbanization with increasingly dense built environments. Several aspects of climate change can make it seem abstract and distant. First, the cross-length definition of climate itself makes conceptualizing climate change involve a considerable degree of abstraction (Stern, 2000). Second, although climate change has many adverse effects on humans, most of its effects will only materialize in the future, which makes it a temporary remote issue (Marx et al., 2007; Gifford, 2011). The remote and abstract characteristics of climate change are embedded in public perceptions that climate change is more relevant in the future than in the present (Leviston et al., 2014; Hanson-Easey et al., 2015; De Guttry et al., 2017). Many scholars have conducted research on how to guide individuals to engage in environmental behavior from the perspectives of environmental significance (Stern, 2000), environmental responsibility (Hines et al., 1987), and pro-environmental behavior (Lee et al., 2020) in order to achieve sustainable development. Citizen environmental behavior is mainly reflected in water and electricity saving, classified recycling of solid waste, green consumption, persuading others to protect the environment, and paying attention to and participating in the formulation of environmental protection policies (Lee et al., 2014). Social cognitive theory states that individual behavior is determined by cognitive and situational factors (Heffernan, 1986). While an individual’s cognition is stable in the short term, with continuous changes in the environment, individual environmental behavior is likely to exhibit “fluctuations.” In general, these studies show that women, middle-aged and elderly people, well-educated people, married people, and urban residents with higher personal income tend to have more environmental awareness, and the motivation for environmental behavior mainly focuses on environmental attitudes, values, social media, information disclosure, and other factors.

(3) Since 2015, relevant studies have begun to focus on sustainable development and evaluating the impact of planned behavior theory and psychological distance theory on citizen environmental behavior. The planned behavior theory states that human behavior is the result of deliberate planning, which includes five elements: attitude, subjective norms, perceived behavior control, behavior intention, and behavior (Liu et al., 2021; Reijonen et al., 2021). With the acceleration of industrialization, an increasing number of countries and individuals have begun to realize that “lucid waters and lush mountains are invaluable assets” and thus have integrated a sustainable development strategy into their own development process. At the latest carbon peak global climate conference, various countries and regions instituted new emission reduction goals to better cope with global climate change. The results are shown in Figure 5.

## Discussion

In the past 10 years, the relevant literature on citizen environmental behavior has increased. The life cycle theory shows that an event will cover gestation to extinction, wherein each stage has different characteristics (Koval et al., 2017). Accordingly, citizen environmental behavior research will also experience a development process from less to more and from rising to falling. This study explores the life cycle of citizen environmental behaviors. According to the literature and development trends, three periods can be identified: infancy, growth, and outbreak. Although it is impossible to accurately predict the future life cycle of citizen environmental behavior, this research remains an important topic and has broad application prospects and theoretical value regarding development trends.

In infancy, theoretical research on this subject was in its early stage, and the average annual number of articles was less than nine, lasting 45 years. During this time, relevant research mainly focused on the impact of environmental reproduction on human environmental behavior (Gifford, 2011). However, during this stage, the research field had certain limitations. Specifically, researchers did not study citizen environmental behavior deeply and did not consider how changes in the ecological environment affect all aspects of human life.

After infancy, research on citizen environmental behavior ushered in a period of steady development and began to focus on the motivation behind citizen environmental behavior from the perspective of psychological distance (Hong and Park, 2018; Maiella et al., 2020). Climate change caused by accelerating urbanization poses a serious threat to human health development (Carmi and Kimhi, 2015). In this context, we studied the motivation of citizen environmental behavior, such as environmental attitude (Haden et al., 2012) and values (Garğarsdóttir et al., 2020), which can better deal with the environment and protect biodiversity. At this stage, more research on citizen environmental behavior was performed from the perspective of psychological distance, and relevant research was developed to some extent. Citizen environmental behavior is affected by both internal and external factors. Internal factors include environmental attitudes, values, environmental beliefs, education level, and income level, and external factors include social norms, social media, social support, environmental pressure, laws, and regulations. In the analysis of the collected literature, we found that some environmental behavior motivation are worth exploring. For example, in the face of climate change, some people started from their personal interests and ignored risk perception; Even though some people recognized the seriousness of climate change, they are unwilling to participate in low-carbon behavior. These motivation are sometimes difficult to explain citizen environmental behavior. In addition, it will have an interactive impact with many aspects such as society, economy, politics and culture, which makes citizen environmental behavior more complex, Summarizing the general motivation of environmental behavior is conducive to the government to formulate targeted policies and guide citizens to make environmental behavior.

In the outbreak period, citizen environmental behavior was further developed from the perspective of psychological distance, during which many scholars began to apply psychological distance theory to study the internal mechanism of citizen environmental behavior, revealing a diversified research trend. In particular, an increasing number of people have begun to pay attention to sustainable development and support policies and regulations related to environmental behavior. However, despite many climate change advocacy activities are conducted, a large number of people are still reluctant to engage in mitigation action (Chen et al., 2020), we must further study how to effectively improve the citizen pro-environmental behavior, including urban ride-sharing (Jin et al., 2020; Zhang et al., 2020), water conservation (Zhuang et al., 2018), public bike-and-ride (Qin et al., 2018), ecotourism (Hwang and Lee, 2018), etc. Existing studies have shown that by publicizing the seriousness of climate change, the public can have a clearer risk perception of climate change and narrow the psychological distance, which helps citizens to make pro-environmental behavior. Technologies narrowing psychology distance includes immersive media, game experience etc. Relevant research is still scarce. In addition, climate change concerns the whole world and is closely related to the fate of mankind. In recent years, the international community has carried out extensive and in-depth cooperation, countries have put forward the goal of energy conservation and emission reduction at the recent world climate conference, putting forward the vision of carbon peak and carbon neutralization, and exploring the establishment of a global climate cooperation mechanism. Future research should focus on global climate cooperation and achieve sustainable development (Saphores et al., 2012; Graves et al., 2013; Greaves et al., 2013; Jones D. A. et al., 2017).

In the past few decades, the concentration of greenhouse gases caused by excessive pursuit of economic development has been increasing. Climate change and increasingly frequent extreme climate events have increasingly threatened human survival and development. Based on this, countries around the world are working together to meet the challenge of climate change, paying attention to environmental protection, increasing energy conservation and emission reductions, and setting emission reduction targets. At present, 135 countries and regions around the world have promised to achieve “carbon neutralization” by the middle of the 21st century. Realizing carbon peak and carbon neutralization involves economy, industry, scientific and technological progress, system and mechanism and so on, which requires long-term, arduous and unremitting efforts. We must take the comprehensive green transformation of economic and social development as the guide and the green and low-carbon development of energy as the key, accelerate the formation of green and low-carbon industrial structure, production mode, lifestyle and spatial pattern, and unswervingly follow the high-quality development road of ecological priority, green and low-carbon. In order to build a sustainable, green and beautiful world, each of us needs to participate, contribute our own strength, advocate a simple, moderate, green and low-carbon lifestyle, and encourage the use of green products for energy conservation and emission reduction. Relevant departments should popularize the use of “Internet plus” to promote green consumption, promote e-commerce enterprises to directly sell or cooperate with entities enterprises to operate green products and services, encourage the use of Internet to sell green products, promote the online trading of second-hand products, and meet the diversified green consumption needs of different subjects. The governments should speed up the construction of urban rail transit, bus lanes and BRT Systems, strengthen the construction of urban slow traffic systems such as bicycle lanes and pedestrian footpaths, promote shared traffic modes such as online car hailing, shared bicycles and car rental, and enhance the effectiveness of green travel incentives. Citizen environmental behavior will be of decisive significance to the realization of carbon peak and carbon neutralization. In the future, we predict that citizen environmental behavior will be more systematically and thoroughly studied in combination with other theories, methods, models, technologies, and policies.

## Conclusion and Future Trends

### Conclusion

In this study, CiteSpace and VOSviewer, two authoritative scientometrics analysis software programs, were used to visually analyze 2,633 articles on citizen environmental behavior in the WOS core database. Based on these articles, the following conclusions were drawn. According to the number of published papers, citizen environmental behavior research has experienced three stages: infancy, growth, and outbreak. After 2006, the number of published research papers showed a rapid growth trend. Currently, this research is experiencing an explosive period, showing that it has broad potential. From the perspective of national distribution, the regional distribution of scientific research forces is uneven in the field of citizen environmental behavior, and the United States, China, Great Britain, and Germany are the top four countries in the field of citizen environmental behavior, accounting for 59.93% of the total literature. Regarding journal distribution, the obtained articles were mainly published in ecology, environmental science, zoology, behavioral science, environmental research, and other relevant journals. After counting the frequency of keywords, we conducted a keyword cluster analysis and summarized three research hotspots of citizen environmental behavior, including “drivers of psychological distance of environmental change perception on citizen environmental behavior,” “the impact of social distance on adolescents’ behavior,” and “the construal level theory and citizen green behavior.” Then, a keyword time zone map was used to clearly show the research content of each development stage, which was used to build models to analyze the impact of environmental reproduction on human environmental behavior in order to achieve strategic objectives, such as improving the environment and focusing on psychological distance theory in the development stage, to determine the motivation behind citizen environmental behavior. Finally, importance was assigned to sustainable development and research regarding the impact of planned behavior theory and psychological distance theory on citizen environmental behavior.

With the rapid development of economy, environmental problems have become a major challenge for sustainable development. Coordinating the balance between economic development and environmental protection has gradually become the consensus of all mankind. Citizen environmental behavior has an extremely important positive impact on promoting environmental protection. There are many related studies on citizen environmental behavior. Starting from the individual level, this paper focused on citizen environmental behavior from the perspective of psychological distance, including the motivation and classification of environmental behavior, and how these factors affect citizen environmental behavior. This paper put forward a new perspective and research direction for the following study, which has certain theoretical significance. Through systematic analysis of the motivation of citizen environmental behavior, such as values, attitudes and knowledge at the individual level, social media, social norms, laws and regulations at the social level, these subdivided influencing factors and their functions will help the government introduce more targeted policies, publicize the risk of climate change, narrow psychological distance, and guide the public to consciously and actively carry out pro-environmental behavior, which has certain practical significance.

### Future Trends

In the past decade, scholars have conducted in-depth research on the impact of psychological distance on citizen environmental behavior and have achieved remarkable results, showing great research potential. This study analyzes the future trends in citizen environmental behavior from the perspective of research direction and scope.

(1) Research on citizen environmental behavior from a single theory has been popularized by a combination of multiple theories. Currently, research on psychological distance mainly includes four dimensions: space distance, time distance, social distance, and hypothesis. However, only a few dimensions of psychological distance were observed in most studies, to better analyze the impact of psychological distance on citizen environmental behavior, studies should analyze the relationship between all dimensions of psychological distance (Spence and Pidgeon, 2010). While every dimension of psychological distance interacts, existing research rarely systematically analyzes the impact of multi-dimensional distance on citizen environmental behavior. To determine citizen environmental behavior, we can analyze the influence mechanism behind citizen environmental behavior in combination with other theories, such as the planned behavior theory and self-determination theory (Botetzagias et al., 2015; Makki et al., 2015).

(2) Further, we can analyze the motivation of citizen environmental behavior and summarize the general motivation of environmental behavior according to its internal relationship. Citizen environmental behavior is affected by both internal and external factors. Internal factors include environmental attitudes, values, environmental beliefs, education level, and income level, and external factors include social norms, social media, social support, environmental pressure, laws, and regulations (Jalu et al., 2019). When social members perceive that the adoption of pro-environment behaviors is justice-enforceable, and then the more proximal the psychological distance they perceive, the more willing they are to adopt pro-environmental behavior, similarly, uneven perceived psychological distance of social members can harm their adoption and the spread of pro-environment behavior (Xu et al., 2020). Increasing global climate change will interact with many aspects, including society, economy, politics, and culture, thereby complicating the motivation of citizen environmental behavior (Wang et al., 2019). Whether these factors affect citizen environmental behavior positively or negatively, or in a proportional function, will become an important topic in the research field. According to the research results, the general motivation for environmental behavior can be summarized.

(3) Additional researches should be conducted on how to narrow the global psychological distance, strengthen the sense of community of human destiny, and explore the establishment of an efficient global climate cooperation mechanism. Climate change is related to global sustainable development, mainly including global warming, acid rain, and ozone layer destruction, which is complex, unbounded, and destructive, and will produce a global “ripple effect.” To improve the international community’s ability to cope with extreme climate change, it is necessary to strengthen the consensus of a “community of human destiny.” Future research may start from the perspective of narrowing global psychological distance (Fox et al., 2020; Gu et al., 2020; Breves and Schramm, 2021), including improving empathy for citizens of disaster-stricken countries in terms of space distance and enhancing awareness of environmental protection through social media in terms of time distance, as well as the hypothesis of a specific quantitative analysis of climate change. Rather than evade their responsibilities, countries must adhere to the guiding principles of multilateral cooperation to jointly address climate change, constantly improve the public’s awareness of environmental protection, and explore the establishment of an efficient global climate cooperation mechanism.

## Data Availability Statement

The datasets presented in this study can be found in online repositories. The names of the repository/repositories and accession number(s) can be found below: https://www.webofscience.com/wos/alldb/basic-search.

## Author Contributions

XF contributed to the analysis and interpretation of data for the study and wrote the first draft of the manuscript. LY designed the framework for this study. JZ contributed to the acquisition of data for this study. All authors approved the final manuscript.

## Conflict of Interest

The authors declare that the research was conducted in the absence of any commercial or financial relationships that could be construed as a potential conflict of interest.

## Publisher’s Note

All claims expressed in this article are solely those of the authors and do not necessarily represent those of their affiliated organizations, or those of the publisher, the editors and the reviewers. Any product that may be evaluated in this article, or claim that may be made by its manufacturer, is not guaranteed or endorsed by the publisher.
